# Addition of 17-(allylamino)-17-demethoxygeldanamycin to a suboptimal caspofungin treatment regimen in neutropenic rats with invasive pulmonary aspergillosis delays the time to death but does not enhance the overall therapeutic efficacy

**DOI:** 10.1371/journal.pone.0180961

**Published:** 2017-07-24

**Authors:** Jeannine M. Refos, Alieke G. Vonk, Marian T. ten Kate, Kimberly Eadie, Henri A. Verbrugh, Irma A. J. M. Bakker-Woudenberg, Wendy W. J. van de Sande

**Affiliations:** Department of Medical Microbiology and Infectious Diseases, Erasmus University Medical Centre, Rotterdam, The Netherlands; Leibniz-Institut fur Naturstoff-Forschung und Infektionsbiologie eV Hans-Knoll-Institut, GERMANY

## Abstract

Caspofungin (CAS) which is used as salvage therapy in patients with invasive pulmonary aspergillosis (IPA) inhibits the 1,3-β-D-glucan synthesis in *Aspergillus fumigatus*. Inhibiting 1,3-β-D-glucan synthesis induces a stress response and in an invertebrate model it was demonstrated that inhibiting this response with geldamycin enhanced the therapeutic efficacy of CAS. Since geldamycin itself is toxic to mammalians, the therapeutic efficacy of combining geldamycin with CAS was not studied in rodent models. Therefore in this study we investigated if the geldamycin derivate 17-(allylamino)-17-demethoxygeldanamycin (17-AAG) was able to enhance the therapeutic efficacy of CAS *in vitro* and in our IPA model in transiently neutropenic rats. *In vitro* we confirmed the earlier demonstrated synergy between 17-AAG and CAS in ten *A*. *fumigatus* isolates. *In vivo* we treated *A*. *fumigatus* infected neutropenic rats with a sub-optimal dose of 0.75 mg/kg/day CAS and 1 mg/kg/day 17-AAG for ten days. Survival was monitored for 21 days after fungal inoculation. It appeared that the addition 17-AAG delayed death but did not improve overall survival of rats with IPA. Increasing the doses of 17-AAG was not possible due to hepatic toxicity. This study underlines the need to develop less toxic and more fungal specific geldamycin derivatives and the need to test such drugs not only in invertebrate models but also in mammalian models.

## Introduction

Invasive pulmonary aspergillosis (IPA), mainly caused by the fungus *Aspergillus fumigatus*, is a difficult to treat, life-threatening fungal infection observed in severely immunocompromised patients. Mortality rates up to 50%-90% are observed in these patients [1]. The standard therapy for IPA remains voriconazole, an inhibitor of the ergosterol synthesis. Unfortunately, voriconazole resistance is emerging necessitating the search for novel therapeutic options [1, 2].

When patients do not respond to voriconazole, caspofungin (CAS) is used as salvage therapy [3, 4]. CAS inhibits the 1,3-β-D-glucan synthesis, an essential component of the fungal cell wall, which provides osmotic stability to fungi and is essential in fungal growth and division [5, 6]. Inhibition of 1,3-β-D-glucan synthesis by the echinocandins causes loss of cell wall integrity and induces an acute stress response [7], in which the chaperone molecule heat shock protein 90 (Hsp90) plays a key role [8–10]. Hsp90 belongs to a group of evolutionarily conserved proteins which can be found in a large group or organisms including humans. In all organisms, including fungi and human, they act as sensors and guardians of cell integrity by preventing mis-folding or aggregation of proteins and triggering adaptive responses under stress conditions [11]. Hsp90 is essential for the survival of eukaryotes, and increased expression of Hsp90 above the level observed in normal tissues is a common feature of human cancers [12]. Therefore Hsp90 is explored as a target for anticancer medication [12]. As such, 17 different Hsp90 inhibitors have entered clinical trials to determine the therapeutic activity against various cancers [13]. Most of these are synthetic inhibitors based on the natural product geldanamycin [13]. The most studied derivatives are 17-dimethyl aminoethylamino-17-demethoxygeldanamycin (17-DMAG) and 17-allylamino-17-demethoxygeldanamycin (17-AAG), which are considerably less toxic than geldanamycin itself. Since Hsp90 plays such an important role in the *A*. *fumigatus* stress response, it was already demonstrated that adding 17-DMAG or 17-AAG to CAS in *in vitro* susceptibility assays, induces synergy against *A*. *fumigatus* [14, 15]. *In vivo*, the therapeutic efficacy of combining geldamycin or one of its derivatives and CAS has hardly been studied. Only one study within the invertebrate *Galleria mellonella* has been published [14]. Detailed data in mammalian models are currently lacking. Within the *G*. *mellonella* model, combination of CAS and geldanamycin resulted in significantly improved survival of larvae infected with a lethal dose of *A*. *fumigatus* compared to mono-therapy treated larvae [14]. However, the invertebrate model does not represent the complexity of the mammalian body. We therefore wondered if the same effect would be obtained in a mammalian host. In the current study, we investigated if combining CAS with geldamycin derivative 17-AAG would enhance the therapeutic efficacy of CAS in our well known animal model of unilateral invasive pulmonary aspergillosis in neutropenic rats [16–23].

## Materials and methods

### *Aspergillus fumigatus* strains

The *A*. *fumigatus* strain EMC01was used in all experiments. This strain is originally isolated from a hematological patient with IPA. To maintain its virulence it was regularly passed through neutropenic rats and maintained on Sabouraud maltose agar slants. To determine the *in vitro* interaction between CAS and 17-AAG, another eight clinical *A*. *fumigatus* isolates from different patients and the *A*. *fumigatus* reference strain ATCC 204305 were included. The clinical strains were isolated from the lower airways of patients with IPA seen admitted to the Erasmus University Medical Center, Rotterdam, the Netherlands in 2005. The strains were maintained on Sabouraud maltose agar.

### *In vitro* susceptibility test

The combination of 17-AAG (LC Laboratories Inc, Woburn MA, USA) and CAS (Cancidas; Merck & Company, Rahway NJ, USA) was investigated for synergism against strain EMC01 and strain ATCC 204305, as well as the other eight clinical *A*. *fumigatus* isolates. Experiments were performed in duplicate using the broth microdilution checkerboard titration technique based on the Clinical and Laboratory Standards Institute (CLSI) methods [24]. The final concentration of the antifungal agents ranged from 4–128 μg/ml for 17-AAG, and from 0.025–128 μg/ml for CAS. MIC endpoints after 48h of incubation at 37°C were determined visually. Also another assessment was used in which the substrate 2,3-bis(2-methoxy-4-nitro-5-[(sulphenylamino)carbonyl]-2H-tretrazolium-hydroxide (XTT) was added as described previously [25]. In this assay, the inhibitory concentration endpoints were defined as the first concentration at which spectrophotometrically 80% or more reduction of mitochondrial dehydrogenase activity occurred. The fractional inhibitory concentration indexes (FICI) were calculated using method one according to Bonapace et al. [26]. In this method, the FIC index is calculated using the concentrations in the first non-turbid (clear) well found in each row and column along the turbidity/non-turbidity interface with the formula FICI = [(MIC_A_ in combination)/MICA] + [MIC_B_ in combination/MIC_B_] and then averaged. For each isolate, FICIs were determined in triplicate. Drug interactions were classified as synergistic (FICI ≤ 0.5), indifferent (0.5 < FICI > 4), or antagonistic (FICI ≥ 4)[24, 27].

### Infection model

The neutropenic rat model of IPA used was described previously [16, 18, 19, 21]. In brief, transient neutropenia in female pathogen albino RP rats (18–25 weeks old, 185–250 grams) was induced by intraperitoneal administration of cyclophosphamide (Endoxan, Baxter, Utrecht, The Netherlands) in doses of 75 mg/kg during five days before fungal inoculation, followed by administration of 60 mg/kg one day before inoculation and 50 mg/kg and 40 mg/kg on days three and seven after inoculation, respectively. A left-sided pulmonary infection was established by intubation of the left main bronchus, while the rats were under general anaesthesia. A cannula was passed through the tube and the left lung was inoculated with 20 μl phosphate buffered saline (PBS) containing 6 ×10^4^ *A*. *fumigatus* conidia of strain EMC01. To prevent bacterial superinfections, rats were given ciprofloxacin (500 mg/L) in their drinking water and teicoplanin intramuscularly in doses 30 mg/kg on day five and day one before fungal inoculation, and 15 mg/kg on days one, three, six, eight and ten after inoculation. During the experiment, the researchers monitored the animals once a day, except for severe infection period, from day three to day seven, than the animals were at least checked every eight hours during the whole experiment and if needed several times a day. Rats were monitored according to a discomfort scale, that is a well-being score in which parameters are scored by appearance (fur coat standing up, extremely pale, temperature reduction, red-rimmed eyes, dirty nose), behaviour (wheezing, gasping, instability), reaction to stimuli and body weight. The extent of the score-scale consists of scoring the parameters; no discomfort (score one), minor (poor (score three); serious (score four) and severe (score five). During the severe infection period the animals have difficulty breathing and may die of the infection. In order to limit the suffering, in case of high discomfort of a seriously ill animal (score four-five), the rats were euthanized by CO_2_. To calculate the average discomfort per group of rats at day three, day ten and day 21, the average discomfort per group was taken. For calculation purposes, rats already died previously during the experiment received score six. If at a certain time all rats had died, the average over that time point was six. Due to the extensive monitoring, the duration of severe distress is usually less than eight hours, and always less than one day.

The experimental protocols used in this study adhered to the rules laid down in the Dutch Animal Experimentation Act and the EU Animal Directive 2010/63/EU. The Institutional Animal Care and Use Committee of the Erasmus University Medical Centre Rotterdam approved the present protocols (permit number: EMC 2692).

### Therapeutic efficacy of antifungal treatment

For the treatment of rats with IPA CAS was diluted in saline and 17-AAG was suspended in 20% cremophor®EL (Sigma-Aldrich Co. LLC, St. Louis MO, USA). CAS at a sub-optimal dose of 0.75 mg/kg and 17-AAG at 1 mg/kg were administered intraperitoneally at 16h after fungal inoculation and from then once daily for ten days. A sub-optimal dosage was chosen to investigate the potential increase in therapeutic efficacy following the addition of 17-AAG. In other groups of animals CAS and 17-AAG were given as monotherapy. Vehicle treated rats served as controls. All groups consisted of 13 rats.

The survival of rats was monitored for 21 days after fungal inoculation. Venous blood was obtained from the tail vein to assess the fungal load in blood on days three and ten by measuring serum galactomannan (GM-index), using a commercially available system according to the manufacturer’s instructions (Platelia *Aspergillus* EIA Platelia *Aspergillus* system of BioRad, Marnes-la-Coquette, France). In addition, from rats with severe disease progression and euthanized, lungs were obtained, fixed in buffered formalin and embedded in paraffin. They were processed for histology using standard techniques. A single cross section of the lung was taken and slides were stained with Grocott to visualize the fungal burden at the site of infection. To describe the burden, the overall fungal mass was described and the density of the fungal mass was assessed at 40x and 200x magnification.

### Toxic side effects of antifungal treatment

To assess renal and hepatic function, serum creatinine (CREAT), blood urea nitrogen (BUN), alanine aminotransferase (ALAT) and aspartate aminotransferase (ASAT) (median ± SD) levels were determined on day three and day ten in animals given mono- or combination therapy. The upper limits of normal were 7.6 mmol/L, 60.2 μmol/L, 86.4U/L and 118.5 U/L for BUN, CREAT, ALAT and ASAT, respectively. They were determined in previous studies [16, 21]. Mild toxicity was defined as parameters levels exceeding the boundary of three times the upper limit of normal, which were 22.8 mmol/L, 180.6 μmol/L, 259.1U/L and 355.5 U/L for BUN, CREAT, ALAT and ASAT, respectively. Severe toxicity was defined as parameter levels exceeding the boundary of five times the upper limit of normal, which were 38.0 mmol/L, 301.0 μmol/L, 431.9 U/L and 592.5 U/L for BUN, CREAT, ALAT and ASAT, respectively [16, 21].

### Statistical analysis

Kaplan-Meier survival curves were generated, and the differences in rat survival rates were assessed by the log rank test (GraphPad Prism 5.0). A value of p<0.05 was considered statistically significant. The quantitative parameters of fungal infection were assessed by using the nonparametric Mann-Whitney U-test (GraphPad Prism 5.0).

## Results

### *In vitro* susceptibility of *A*. *fumigatus* strains

As shown in Table 1, all tested *A*. *fumigatus* strains were inhibited in growth by 128 μg/ml of CAS. No inhibitory effect was found for 17-AAG (MICs > 128 μg/ml), except for strain Af42, which had a MIC of 128 μg/ml. A synergistic interaction (FICI ≤ 0.5) was observed when 17-AAG was combined with CAS, for the EMC01 strain, the ATCC strain and four clinical isolates (Af45, Af46, Af47 and Af48). An indifferent effect was observed for the remaining clinical isolates, Af38, Af41, Af42 and Af44.

**Table 1:** FICI of 17-AAG and CAS against *A*. *fumigatus*, reference strains and clinical isolates.

Strain	Agent	MIC (ug/ml) of each agent alone	FICI*	Outcome
**ATCC**	17-AAG	>128	0.4	synergistic
	Caspofungin	128		
**EMC01**	17-AAG	>128	0.5	synergistic
	Caspofungin	128		
**Af38**	17-AAG	>128	1	indifferent
	Caspofungin	128		
**Af41**	17-AAG	>128	1	indifferent
	Caspofungin	128		
**Af42**	17-AAG	128	2	indifferent
	Caspofungin	128		
**Af44**	17-AAG	>128	0.9	indifferent
	Caspofungin	128		
**Af45**	17-AAG	>128	0.3	synergistic
	Caspofungin	128		
**Af46**	17-AAG	>128	0.3	synergistic
	Caspofungin	128		
**Af47**	17-AAG	>128	0.4	indifferent
	Caspofungin	128		
**Af48**	17-AAG	>128	0.4	synergistic
	Caspofungin	128		

*FICI is calculated using the concentrations in the first non-turbid (clear) well found in each row and column along the turbidity/non-turbidity interface with the formula FICI = [(MIC_A_ in combination)/MICA] + [MIC_B_ in combination/MIC_B_] and then averaged. FICI calculated before and after adding XTT was identical.

### *In vivo* therapeutic effect of 17-AAG in combination with CAS on survival of rats with IPA

**Fig 1:**
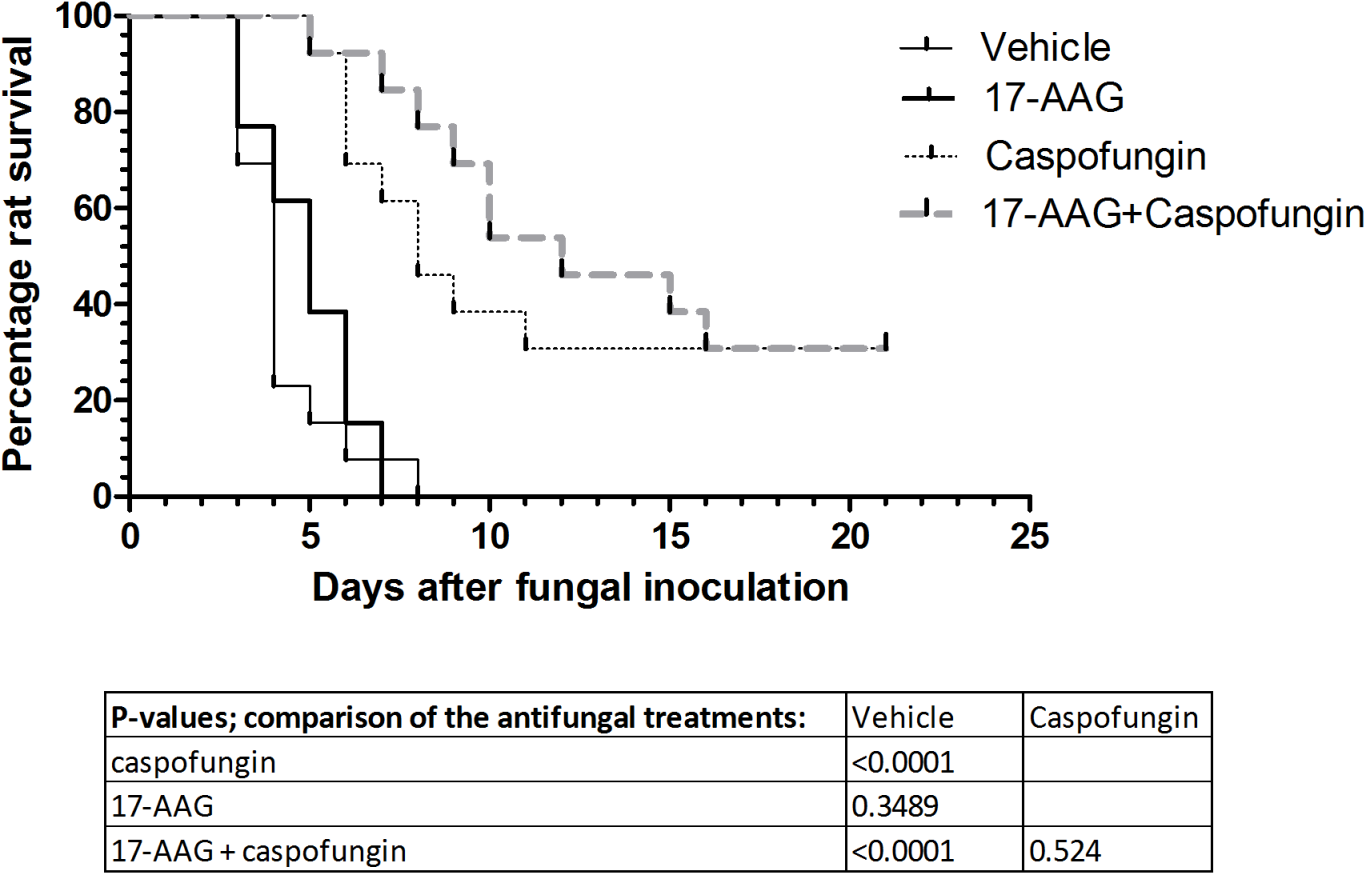
Survival of neutropenic rats with invasive pulmonary aspergillosis during treatment with 17-AAG and/or Caspofungin. Treatment with 17-AAG 1 mg/kg or Caspofungin 0.75 mg/kg alone or in combination was started 16h after fungal inoculation at day 0 and was administered intraperitoneally to rats (n = 13 per treatment group) once daily for ten days. The survival of rats was monitored for 21 days after fungal inoculation.

As shown in Fig 1, all vehicle-treated control rats died between day three and day eight after fungal inoculation. Treatment with CAS alone resulted in a rat survival rate of 31%, as expected. Treatment with 17-AAG alone did not improve rat survival compared to the vehicle treated rats (*p* = 0.35). Treatment with the combination of 17-AAG and CAS did not result in a significantly increased rat survival compared to rat survival obtained with CAS monotherapy (*p* = 0.52). However, a delay was observed in the median time to death in rats treated with the combination of 17-AAG and CAS (Fig 1).

To evaluate if a higher dose of 17-AAG in combination with CAS would enhanced the therapeutic effect, increased dosages of 17-AAG at 5 mg/kg/day or 20 mg/kg/day in combination with CAS were administered. Unfortunately, these higher 17-AAG dosages were not tolerated by the rats and resulted in renal and hepatic toxicities (data not shown).

### *In vivo* toxic side effects of the different treatment schedules

In order to investigate whether 17-AAG administration was well tolerated by the rats, we investigated the renal and hepatic functions of the treated animals by determination of BUN, CREAT, ALAT and ASAT levels in serum on day three and day ten of administration. As shown in Table 2, neither CAS 0.75 mg/kg monotherapy nor 17-AAG 1 mg/kg monotherapy nor the combination therapy resulted in altered high levels as these parameters were near the upper limit of normal.

**Table 2:** Renal and hepatic functions of neutropenic rats with IPA.

Antifungal treatment	BUN (mmol/L)		CREAT (μmol/L)		ALAT (U/L)		ASAT (U/L)		Average discomfort**		
	day 3	day 10	day 3	day 10	day 3	day 10	day 3	day 10	Day 3	Day 10	Day 21
										
Vehicle	8.9 (± 3.1)	ND	89.6 (± 42.3)	ND	48.0 (± 14.8)	ND	95.2 (± 20.5)	ND	3.92	6	6
Caspofungin	5.0 (±1.2)	8.1 (±0.4)	55.5 (±24.0)	58 (±6.1)	45.5 (±5.2)	55.0 (±4.9)	122.5 (±28.2)	122.5 (±9.7)	2.58	4.50	5.00
17-AAG	8.8 (± 2.7)	ND	99.4 (± 50.4)	ND	33.4 (± 6.8)	ND	75.8 (± 26.2)	ND	3.15	6	6
17-AAG + Caspofungin	8.4 (± 3.9)	8.6 (±3.9)	96.6 (± 47.1)	50.6 (±47.7)	44.6 (± 27.1)	(48.4±27.1)	98.6 (± 207.6)	168.6 (±207.7)	2.31	3.85	4.77
Upper limit of normal *	7.6		60.2		86.4		118.5		NA		
Mild toxicity*	22.8		180.6		259.1		355.5		NA		
Severe toxicity*	38.0		301.0		431.9		592.5		NA		

ND, the renal and hepatic functions could not be determined as all rats were severely ill and had to be euthanized.

NA, not applicable

*The upper limit of normal and the mild toxicity and severe toxicity boundaries were determined in previous studies [16, 21] in the same rat strain.

** The discomfort scale ranges from 1–5; rats had no discomfort (score 1), mild (score 2), poor (score 3), serious (score 4) and severe (score 5). For calculation purposes, all animals who died received the number 6. So if the average discomfort states 6, this means that all animals are death at that time point.

### *In vivo* therapeutic effect of 17-AAG in combination with CAS on fungal burden in blood of rats with IPA

**Fig 2:**
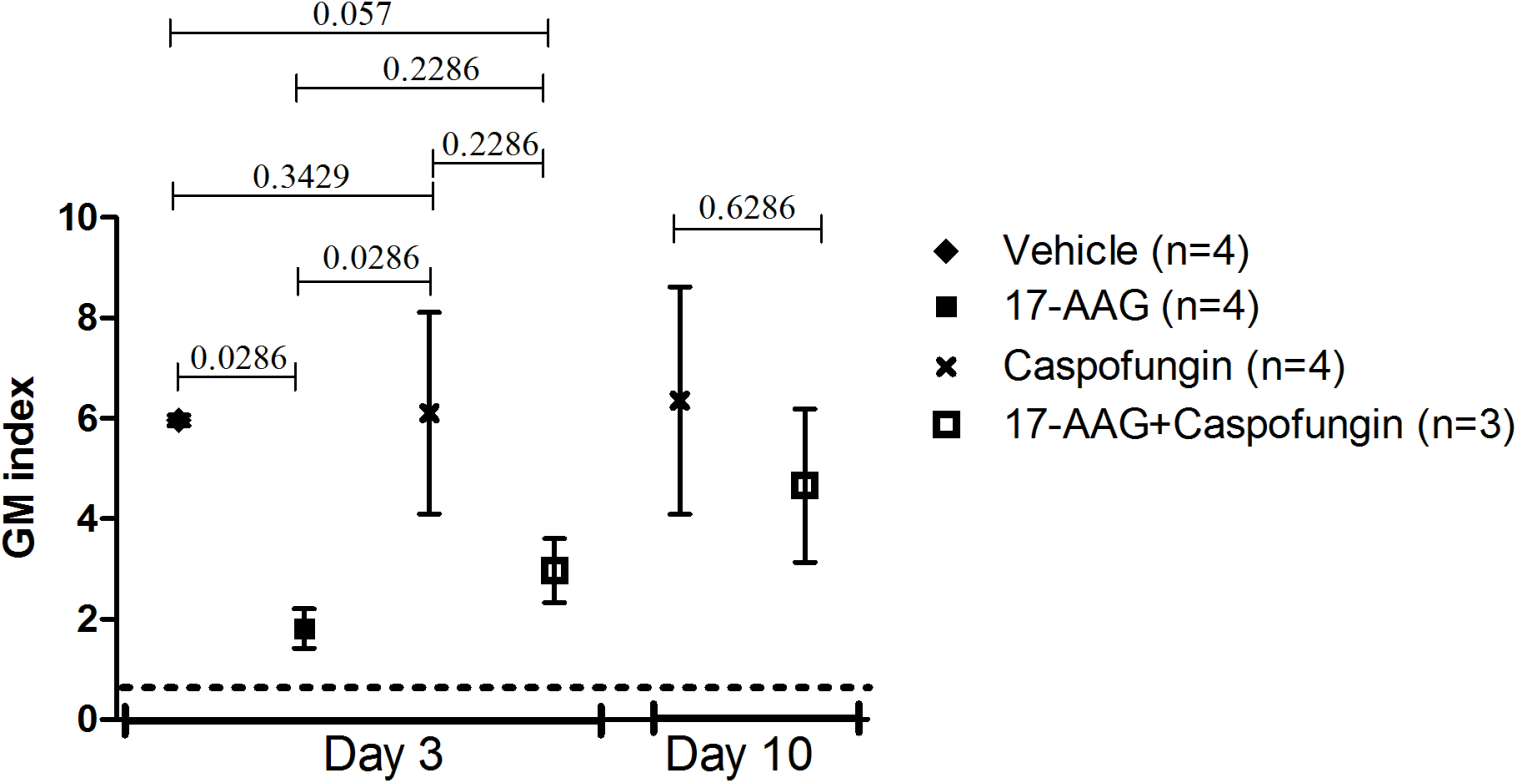
GM-index in blood of neutropenic rats with invasive pulmonary aspergillosis during treatment with 17-AAG and/or Caspofungin. Black diamond (♦): vehicle treatment, black square (■): monotherapy 17-AAG, cross (**x**): monotherapy Caspofungin, open square (□): combination therapy of 17-AAG and Caspofungin. Data were generated by determining the Galactomannan (GM)-index with the Platelia assay for three to four rats per group. Errors bars represent SEM. According to the manufacturer’s manual, GM-index of >0.5 is considered positive for *A*. *fumigatus*.

A mean GM-index of 5.96 (range 5.8–6.3) was observed in the vehicle control rats on day three. The CAS monotherapy group had a mean GM-index of 6.11 (range 2.83–11.93), indicating that CAS at this dose did not reduce fungal burdens. In contrast on day three of therapy a 2–3 fold lower GM-index (mean 1.82, range 1.09–2.56) was observed in 17-AAG treated rats (Mann-Whitney, p<0.0286) compared to the vehicle treated rats (Fig 2). On day ten GM-index increased in the 17-AAG+CAS treated rats surviving to day ten (mean 4.66, range 1.68–6.75). However, the galactomannan levels remained insignificantly below those observed in the CAS monotherapy rats surviving day ten (mean 6.36, range 1.91–12.08)(Fig 2).

**Fig 3:**
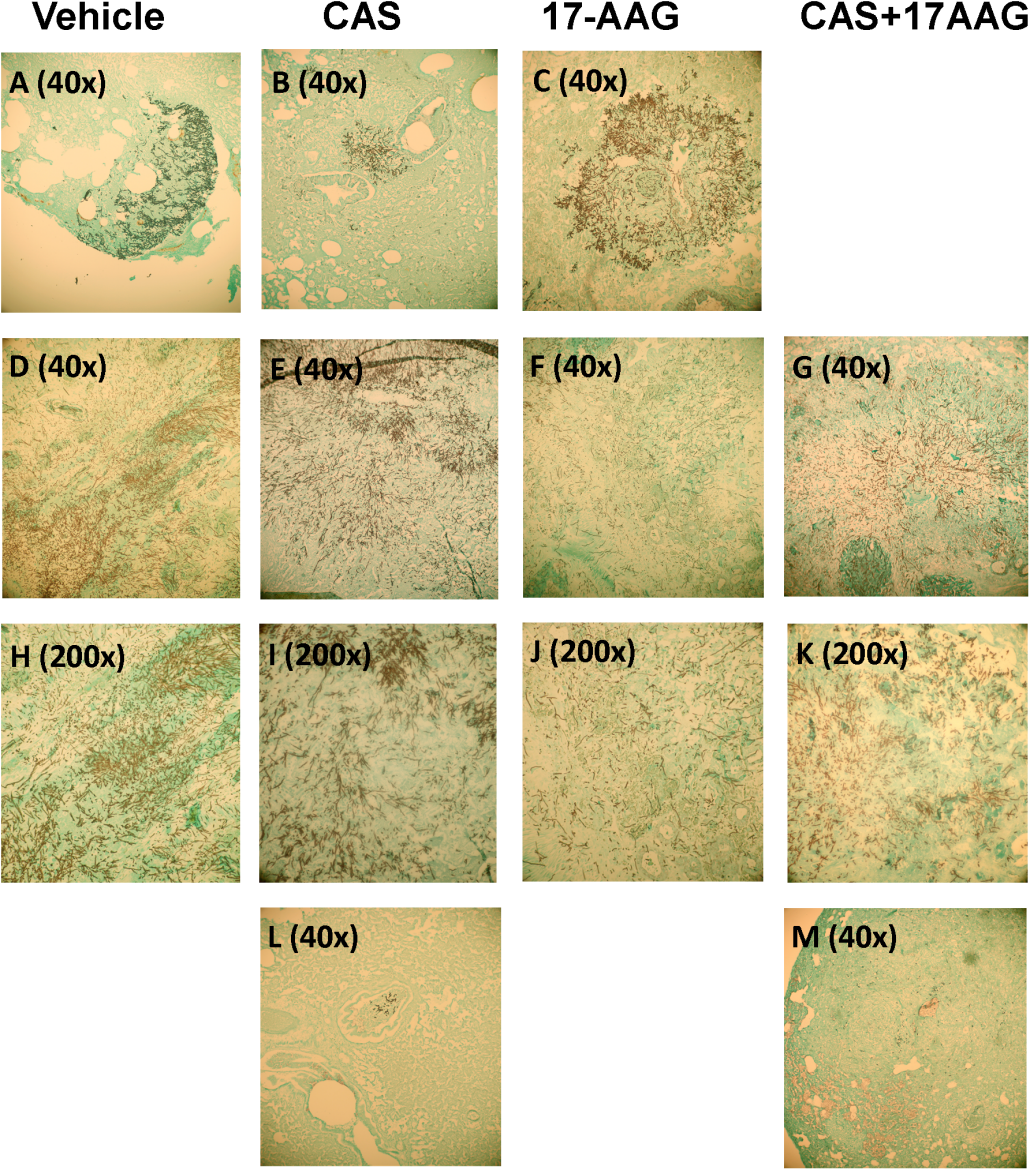
Lung tissue grocott staining. Panels A, B, C, D, E, F, G, L, M (40x), H, I, J and K (200x) show *Aspergillus fumigatus* infected lung tissues. Panels A, D and H were treated with the vehicle. Panels B, E, I and L were treated with CAS monotherapy. Panels C, F and J were treated with 17-AAG monotherapy. Panels G, K and M were treated with CAS and 17-AAG combination therapy. A: *A*. *fumigatus* focus after vehicle treatment at day four (40x magnification). B: *A*. *fumigatus* focus after CAS treatment at day four (40x magnification). C: *A*. *fumigatus* focus after 17-AAG treatment at day three (40x magnification). D: *A*. *fumigatus* focus after vehicle treatment at day five (40x magnification). E: *A*. *fumigatus* focus after CAS treatment at day eight (40x magnification). F: *A*. *fumigatus* focus after 17-AAG treatment at day six (40x magnification). G: *A*. *fumigatus* focus after CAS and 17-AAG treatment at day ten (40x magnification). H: *A*. *fumigatus* focus after vehicle treatment at day five (200x magnification). I: *A*. *fumigatus* focus after CAS treatment at day eight (200x magnification). J: *A*. *fumigatus* focus after 17-AAG treatment at day six (200x magnification). K: *A*. *fumigatus* focus after CAS and 17-AAG treatment at day ten (200x magnification). L: *A*. *fumigatus* focus after CAS treatment at day 21 (200x magnification). M: *A*. *fumigatus* focus after CAS and 17-AAG treatment at day 21 (200x magnification). In the lung tissue of the infected rats, normal morphology of alveoli is lost. Fungal hyphae of *A*. *fumigatus* are clearly seen and coloured black by the Grocott staining. On day three (17-AAG treated rats) and day four (vehicle treated rats) similar fungal foci are seen (A and C). The fungal focus in CAS treated rats seems smaller. On days five, six, eight and ten (D, E, F, G, H, I, J and K) the fungus has been disseminated throughout the lung. However, in the 17AAG treated rat at day six (F, J) the fungal load is seems less dense compared to the vehicle rat at day five (D, H) or the CAS treated rat at day eight (E, I) or the CAS and 17-AAG treated rat at day ten (G, K). At day 21, small fungal foci were seen in CAS treated rats (L) but not in CAS and 17-AAG treated rats (M).

Histopathology of the lung tissue (Fig 3; Grocott stain 4x-20x) shows that there was a small difference in size of the fungal foci between a severely ill vehicle treated rat euthanized on day four (Fig 3A) and a CAS treated rat on day four (Fig 3B): the focus of the CAS treated rat was smaller. No visual difference in fungal burden was observed between the vehicle treated rat (Fig 3A) and a 17-AAG treated rat euthanized on day three (Fig 3C). However, visual comparison of a severely ill vehicle treated rat euthanized on day five (Fig 3D and 3H), a severely ill CAS treated rat euthanized on day eight (Fig 3E and 3I), a severely ill 17-AAG treated rat euthanized on day six (Fig 3F and 3J) and a severely ill CAS and 17-AAG treated rat euthanized on day ten (Fig 3G and 3K) yielded a difference in fungal load in lung tissue. In all rats, the fungal hyphae were already disseminated resulting in a lung infection. However, in the vehicle, CAS mono- and the 17-AAG+CAS treated rat the fungal load was much denser compared to the 17-AAG monotreated rat. On top of that, when the lungs of the surviving rats were compared on day 21, it was noted that only in the CAS mono-therapy small fungal foci remained. In the 17-AAG+CAS treated rats, no fungal foci were found.

## Discussion

To determine the efficacy of a novel therapeutic regimen, it is important to tests it’s efficacy in models in which the features observed in humans are reproduced [28]. Our rat model, characterized by prolonged severe granulocytopenia, inoculation through the respiratory route, fungal broncho- and angio-invasion and dissemination of the fungus from the lung to other organs, closely mimics the pathology observed in human IPA [29]. Within this model, it was possible to study the therapeutic efficacies of voriconazole, CAS and anidulafungin in human pharmacokinetic equivalent dosages, further improving the predictive value of this model [16, 17, 21]. We therefore used this rat model to determine if the therapeutic efficacy of the combination geldamycin and CAS observed in the invertebrate *G*. *mellonella* model [14] could be confirmed in a model mimicking the human disease more closely [16–23]. Although Cowen et al. demonstrated therapeutic efficacy when CAS was combined with Hsp90 inhibitor geldamycin, in their *G*. *mellonella* larvae infected with a lethal dose of *A*. *fumiatus*, we could not confirm this result in our unilateral IPA model in rats. The difference in outcome could have been the result of the difference of host, but also to the difference in the drugs used in combination. Due to the known hepatoxicity of geldanamycin for mammals [30] we used its 17-AAG derivative, which differed from the experimental set-up of Cowen *et al* in the *G*. *mellonella* model [14]. Furthermore, the dosages that were used by Cowen in her larval model, greatly exceed the dosages which can be used in mammalian models without toxic side effects. In the larval model dosage as high as 50 mg/kg geldanamycin were tolerated. In our study in rats, the maximum tolerated dose was 1 mg/kg 17-AAG, whilst a five-fold higher dose of 17-AAG resulted in severe toxicity, limiting further studies using higher dosages of 17-AAG. In our study, we did not investigate doses in the range between 1–5 mg/kg, which might increase rat survival without toxic side effects.

The toxic side effects of 17-AAG as found in our rat model, were also reported by other investigators in mammalian models e.g. mice and dogs; also in these animal models the hepatoxicity and galbladder toxicity as well as renal failure were the dose limiting factors [31]. In addition to hepatic toxicity, the effect of 17-AAG on the immune system of the rat probably might also play a role as demonstrated by other investigators. Geldanamycin was shown to be able to interfere with the maturation of dendritic cells, which resulted in lowered proliferation of T-cells and lower pro-inflammatory cytokine production [32, 33]. Pro-inflammatory cytokines are crucial in the protective immunity against *A*. *fumigatus*. Since our rats already had an impaired host defence, weakening the immune system even further with a geldanamycin derivative might have contributed in the relatively low maximum tolerated dose found in our neutropenic rat model, compared to the published maximum tolerated doses in other mammals with normal functioning immune systems. Although the effect of geldanamycin derivatives on the immune system of *G*. *mellonella* larvae has been not studied, it is possible that toxic side effects will be less in view of their relatively simple immune system consisting of hemocytes and antimicrobial peptides [34], thereby allowing higher concentrations of geldanamycin and its derivatives to be used without any effect on the immune system.

Although the treatment with 17-AAG at 1 mg/kg did not enhance the survival of rats with IPA, we investigated whether this treatment resulted in a decrease in fungal growth in blood or lungs of IPA rats. The fungal load was assessed in terms of concentrations of GM in serum or histopathology of the lung. The detection of GM in serum mainly reflects the presence of an active infection [16]. We observed a similar GM index in the CAS treated rats compared to the vehicle treated rats during infection on day three. However, the fungal focus in the lung of CAS treated rats seemed smaller than the fungal focus of vehicle treated rats on day four. One explanation could be that the high GM-index in the rats receiving CAS is due to the detachment of the galactomannan chains from the β-glucan polymers in the presence of CAS as found by some studies [35]. These detached polymers are released in the blood and thereby resulting in a high GM-index, even when the fungal focus is still small. In contrast, monotherapy 17-AAG resulted in a lower GM-index compared to the vehicle treatment (*p* = 0.0286) on day three. Within the tissue itself, no obvious difference in fungal burden was observed histologically on day three when 17-AAG treated rats were compared to vehicle treated rats at day four. Only at days five, six, eight and ten a difference was noted. In 17-AAG treated rats (day six), the fungal foci were less dense compared to vehicle (day five), CAS (day eight) or 17-AAG and CAS (day ten) treated rats. This indicates, that the observed differences in GM index in the serum could be due to the fact it was not properly released from the cell wall in the 17-AAG treated rats. 17-AAG inhibits HSP90 and low expression of HSP90 results in a low level of calcineurin in fungal cells [36–38]. The importance of calcineurin in the release of GM by *A*. *fumigatus* strains was demonstrated by Mennink-Kersten *et al*. She showed *in vitro*, that an *A*. *fumigatus* strain lacking calcineurin was unable to release GM due to cell wall abnormality, instead GM was retained within the cell wall at concentrations ten- fold higher compared to the wild-type strain [39]. Looking at the survival of CAS-, 17-AAG, CAS and 17-AAG and vehicle treated rats it seemed that the GM index did not predict the severity of the disease in the lung, histopathology was more informative. Unfortunately, the lungs used for the histology comparisons were obtained from animals who had to be euthanized due to severe distress of the fungal infection. This might not necessarily represent the burden in the lung of all animals within that group. Furthermore this also resulted in a day difference for each pair of comparison, which means that there is a day difference in the evolution of the disease. It also limited the number of rats to be used to only one rat per group per time point. The only point in which we could make a clear comparison between the lungs of the different treatment groups was on day 21 in which the lungs of the surviving rats treated either with CAS monotherapy or 17-AAG+CAS were compared. At that time point it apperared that only in the CAS monotherapy treated rats, small fungal foci were still present. They were absent in the 17-AAG+CAS treated rats, implicating that combining a Hsp90 inhibitor with CAS might still be a feasible approach to clear the infection in a shorter time frame. To further investigate the possibilities of targeting Hsp90 in *A*. *fumigatus* infection as an antifungal strategy, efforts need to be taken to develop drugs which target the fungal Hsp90 more specifically in order to reduce potential toxic side effects for the mammalian host. In the past, such an inhibitor was already designed and tested. This inhibitor was the human recombinant antibody Mycograb directed against *Candida* Hsp90. This antibody was specifically designed to work in combination with antifungal agents and entered a multinational phase II clinical trial [40]. Despite these promising results, Mycograb was never marketed, due to batch-to-batch inconsistencies in the composition of the product leading to safety concerns [40].

Although in the present study a beneficial effect in terms of 21-day increased survival of neutropenic rats with IPA could not be demonstrated by combining the Hsp90 inhibitor 17-AAG with CAS, the successful combinations of geldanamycin and CAS in invertebrate hosts and the successful combinations of mycograb with liposomal amphotericin B or CAS in murine models, still indicate that combining Hsp90 inhibitors with antifungal agents might open new ways to enhance antifungal efficacy. Efforts are needed to design specific inhibitors of fungal Hsp90 resulting in reduction of toxic side effects. In addition, next to invertebrate models, mammalian models need to be included to assess the clinical potency of these drugs.
